# Quality of life and its influencing factors among breast cancer patients at Tikur Anbessa specialised hospital, Addis Ababa, Ethiopia

**DOI:** 10.1186/s12885-022-09921-6

**Published:** 2022-08-17

**Authors:** Mikiyas Amare Getu, Changying Chen, Panpan Wang, Eva Johanna Kantelhardt, Adamu Addissie

**Affiliations:** 1 The First Affiliated Hospital of Zhengzhou University, Zhengzhou, China; 2 School of Nursing and Health, Zhengzhou University, Zhengzhou, China; 3 Global Health Group, Martin-Luther-University, Halle (Saale), Germany; 4 Institute of Medical Epidemiology, Biostatistics and Informatics, Martin-Luther-University, Halle (Saale), Germany; 5 Department of Gynecology, Martin-Luther-University, Halle (Saale), Germany; 6 School of Public Health, Department of Preventive Medicine, College of Health Sciences, Addis Ababa University, Addis Ababa, Ethiopia

**Keywords:** Breast cancer, EORTC QLQ-BR45, EORTC QLQ-C30, Quality of life, Ethiopia

## Abstract

**Background:**

Quality of life (QoL) has become an important measure for evaluating cancer patients’ treatment and prognosis. Breast cancer patients are at an increased risk of experiencing poor QoL during active treatment of cancer. This study aimed to assess QoL and it’s influencing factors among breast cancer patients using the newly updated breast cancer specific tool of the European Organisation for Research and Treatment of Cancer EORTC Breast Cancer Specific Quality of Life Questionnaire QLQ-BR45.

**Methods:**

An institutional based crossectional study was conducted with 248 breast cancer patients at Tikur Anbessa Specialized Hospital (TASH). Descriptive statistics, one-way analysis of variance (ANOVA), and linear regression were used to describe and analyze the data.

**Results:**

The participant’s Global health status/QoL mean score was 65.6. Among the functional scales, future perspective scored the lowest (57.1, SD ± 37.3). The highest mean score on the symptom scales/items were financial difficulties (50, SD ± 38.6), followed by appetite loss (37.4, SD ± 36.4) and fatigue (34.3, SD ± 27.1) while the lowest symptom score was diarrhoea (6.4 ± 18.4). EORTC QLQ-BR45, future perspective (mean = 57.1, SD ± 37.3) and upset by hair loss (41.8, SD ± 34.6) were the most affected functioning and symptoms scales respectively. An increased stage of tumor was associated with more pain (*P* = 0.041), appetite loss (*P* = 0.042), and arm symptoms (*P* = 0.003). Patients who had no comorbidity had better physical (*P* < 0.001), cognitive (*P* = 0.013), and social (*P* = 0.009) function.

**Conclusion:**

These specific functional scales and symptoms should be assessed individually to address unmet needs. Clinicians could design psychosocial interventions to improve these function and to reduce symptoms.

**Supplementary Information:**

The online version contains supplementary material available at 10.1186/s12885-022-09921-6.

## Introduction

Worldwide, an estimated 19.3 million new cancer cases and almost 10.0 million cancer deaths occurred in 2020. The global cancer burden is expected to be 28.4 million cases in 2040, a 47% rise from 2020 [1]. Breast cancer was the most commonly diagnosed cancer and the leading cause of cancer death among women worldwide in 2020. The age-standardized incidence and mortality rate of female breast cancer in east Africa was 33 and 17.9/100,000 person-years respectively in 2020 [2]. Breast cancer is the leading type of cancer in Ethiopia with an estimated 16,133 new cases annually [1].

Quality of life (QoL) is an individual’s perception of their life in relation to their goals, expectations, standards and concerns. It is a complex concept that includes an individual’s physical health, psychological health, personal beliefs, social interaction and its relationship to their environment [3]. In the field of oncology, patients’ QoL has become a major objective of cancer care. It has been shown that QoL assessment is helpful to predict treatment response and prognosis [4–6]. Several studies have shown that a better QoL measure is associated with longer survival of patients’ with various types of cancer [7–9]. Therefore, it is necessary to assess QoL and its predictors continuously and periodically from diagnosis to survivorship to get insight into patient management and care [10]. Various studies have shown that women are at increased risk of experiencing poor QoL during the active treatment and survivorship phases of cancer care [8]. Breast cancer patients undergoing chemotherapy have poor QoL [7, 11]. Although, breast cancer is the leading cause of morbidity and mortality in Ethiopia, to our knowledge there was no adequate study done to assess QoL and its influencing factors among breast cancer patients. Only two studies were conducted with some limitations. One was completed among breast cancer patients undergoing chemotherapy [12] and the other was done among breast cancer patients during and after systemic therapy [13]. This study used the global health status scale as a dependent variable, despite the presence of other functional and symptoms scales. In the present study, QoL was measured using European Organization for Research and Treatment of Cancer Quality of Life Questionnaire-Core30 (EORTC QLQ-C30) [14] and the updated breast cancer specific tool (EORTC QLQ-BR45) [15]. The EORTC QLQ-C30 is a core questionnaire that assesses the QoL of cancer patients [14]. EORTC QLQ-BR45 is a breast cancer specific module that is used in combination with the EORTC QLQ-C30 core questionnaire [15]. One previous study were conducted using the former EORTC QLQ-BR23 breast cancer specific tool [16].

Major advances have been made in breast cancer diagnosis and treatment since the tool was developed; thus, the EORTC QLQ-BR23 needed to be updated.

This study aims to assess for the first time, quality of life and its influencing factors among breast cancer patients in Addis Ababa using the newly updated breast cancer specific (EORTC QLQ-BR45) and general (EORTC QLQ-C30) instruments.

## Materials and methods

### Study design and setting

A cross-sectional study was conducted from January 1 to March 30, 2021 at Tikur Anbessa Specialised Hospital (TASH) oncology centre in Addis Ababa, Ethiopia. The hospital is a tertiary level teaching hospital and the only radiotherapy centre in the country [17].

### Participants

All female breast cancer patients in any stage of the disease and who had no recurrence were included. All patients were aged 18 years and above who visited TASH during the study period. The participants were receiving or had previously received curative treatment. Patients with psychiatric problems or cognitive impairment who were unable to understand or complete the interview questionnaires were excluded. In addition, patients with other severe illnesses, coexisting malignancies were excluded from the study.

### Measurement

#### Socio-demographic characteristics

Age, educational status, religion, residence, marital status, occupation, and monthly income.

#### Clinical characteristics

Stage at diagnosis, comorbidity, admission status, and previous treatment taken.

#### Quality of life

It was measured by the Amharic version of EORTC QLQ-C30 and EORTC QLQ-BR45 Amharic version.

EORTC QLQ-C30 is a core questionnaire that used to assess QoL of cancer patients [14]. It consists of five functional scales (physical, role, cognitive, emotional, and social), three symptom scales (fatigue, pain, nausea and vomiting), and a Global Health Status (GHS)/ QoL scale. The other single items record symptoms include (dyspnoea, appetite loss, sleep disturbance, constipation, and diarrhoea), as well as financial difficulties [14]. The questionnaire was valid and reliable to assess QoL in cancer patients in Ethiopia [18].

EORTC QLQ-BR23 is breast cancer specific module developed in 1996 to assess breast cancer related specific symptoms [19]. It consists of 23 items which incorporates four functional scales/items (body image, sexual functioning, sexual enjoyment, and future perspective) and four symptom scales (systemic therapy side effects, breast symptoms, arm symptoms, and upset by hair loss) [19]. However, since 1996 the advancement in the diagnostics and therapeutics of breast cancer has brought a change that requires EORTC QLQ-BR23 update. Because, the original EORTC QLQ-BR23 cannot be able to cover many important QoL issues and potential side effects new treatment. Thus, the previous breast cancer specific module was updated to EORTC QLQ-BR45 [15]. EORTC QLQ-BR45 is a newly updated breast cancer specific tool developed by EORTC Quality of Life Group (QLG) which is used in combination with the EORTC QLQ-C30 [15]. The newly updated version has incorporated additional 22 items: target symptom scale (20 items) and breast satisfaction scale (2 items). The target symptom scale can be divided into three subscales: endocrine therapy, endocrine sexual and skin/mucosa scale. It is used to assess breast cancer specific symptoms caused by the disease and its newer therapeutic options. The breast satisfaction scale used to assess patient’s satisfaction to the appearance of skin of affected breast and the cosmetic effect of surgery [15]. The validity and reliability of the tool were assessed in our previous study, and its Amharic version was found to be valid and reliable to measure QoL in breast cancer patients. The Cronbach’s α coefficient was 0.80 and the test-retest reliability coefficient was 0.77 for all domains. The item content validity index (I-CVI) ranged from 0.83 to 1.

### Scoring procedures of QoL

The item scoring procedure for the EORTC QLQ-C30 and the EORTC QLQ-BR45 was managed according to the EORTC QLQ-C30 scoring manual [20].

The EORTC QLQ-C30 and EORTC QLQ-BR45 are rated on a four-level Likert scales response system, from 1 “not at all” to 4 “very much.” Except for the EORTC QLQ-C30 GHS items, Q29 and Q30, a seven-level Likert scale is used, from 1 “very poor” to 7 “excellent” [20]. In this study, the raw scores for both EORTC QLQ-C30 and EORTC QLQ-BR45 scales and single item measures were transformed into scores ranging from 0 to 100. A high scale score represents a higher response level. Thus, a high score for a functional scale represents a high/ healthy level of functioning; a high score for the GHS/QoL represents a high QoL, but a high score for a symptom scale/item represents a high level of symptomatology/ problems [20].

For all scales, the raw score (RS) is the mean of the components items:

1 $$\text{Raw score}=\mathrm{RS}=\frac{\boldsymbol{I1}+\boldsymbol{I2}+\dots +\boldsymbol{In}}{\boldsymbol{n}}$$

Apply the linear transformation to 0–100 to obtain the score S,

2 $$\text{Functional scale}:\mathrm{S}=\left\{\mathbf{1}-\frac{\left(\boldsymbol{RS}-\mathbf{1}\right)}{\boldsymbol{range}}\right\}\ast \mathbf{100}$$

3 $$\text{Symptom scale and Global health status}/\mathrm{QoL}:\mathrm{S}=\left(\frac{\boldsymbol{RS}-\mathbf{1}}{\boldsymbol{range}}\right)\ast \mathbf{100}$$

### Sample size

Two hundred forty breast cancer patients who visited the hospital during the data collection period, and who were willing to participate in the study, were included.

### Data collection procedures

Data was collected by using self-administered questionnaire filled by participants. The socio-demographic characteristics were self-reported by the participants whereas; the clinical characteristics were extracted from the patient’s medical record by trained clinical nurses. Written informed consent was obtained from study participants after providing information about the study. To ensure good quality of data collection, an investigator was reviewing the filled questionnaires throughout the data collection period. If missing values were found, the questionnaire was returned to the patients for completion.

### Statistical analysis

Data was entered, cleaned, and coded into Epidata 4.6 software and exported to Statistical Package for the Social Science (SPSS version 25) for analysis.

Descriptive statistics (frequencies, mean, and standard deviation) were used to describe socio-demographic data such as; age, marital status, educational status, place of residence, and clinical data such as; stage of tumor, comorbidity, and type of treatment. Independent t-test and one-way ANOVA were employed to determine whether differences in the mean score of QoL as measured by the functional and symptom scales of EORTC QLQ-C30 and EORTC QLQ-BR45 across socio-demographic and clinical variables of the participants were significant.

Variables with a *p* value ≤0.2 on correlation analysis were taken in to account in the multivariable linear regression model [21] to assess the predictors of EORTC QLQ-C30 and EORTC QLQ-BR45 scales. Multivariable linear regression analysis permits the study of multiple independent variables at the same time, with adjustment of their regression coefficients for possible confounding effects between variables [22]. Results of the multivariable linear regression analyses were expressed by B (beta) and the total amount of variance explained by the models in R squared. The *P*-value less than or equal to 0.05 were considered significant.

## Results

### Socio-demographic and clinical characteristics

Among a total number of 248 breasts cancer patients, 240 patients agreed to participate in this study with a total response rate 96.8%. The average age of the participants was 44.7 years (SD ± 11.2 years). A majority of the participants (57.9%) were married, Orthodox Christian (59%) and resided in urban area (76.6%).

Most of the study participants (40.8%) had a stage III tumours. Of the participants, 201 (83.8%) had no other illnesses or comorbidities, and 96.7% of participants were under active treatment follow-uThe remaining 3.3% of the particpants had surgery and waiting for the other treatment plan (Table 1).

**Table 1:** Socio-demographic and clinical characteristics of breast cancer patients at TASH, Addis Ababa, Ethiopia, 2021 (*N* = 240)

Variables	Frequency	Percentage
**Age (years)**		
Mean (SD)	44.7 (11.2)	
Median	42.5	
Range	23–85	
**Educational status**		
No education	75	31.3
Primary school education	51	21.3
Secondary school education	57	23.8
Above secondary school education	57	23.8
**Religion**		
Orthodox Christianity	142	59.2
Muslim	41	17.1
Protestant	50	20.8
Catholic	4	1.7
Other ^a^	3	1.3
**Occupational status**		
Housewife	135	56.3
Government employed	35	14.6
Farmer	25	10.4
Merchant	15	6.3
Daily labourer	13	5.4
Other ^b^	17	7.1
**Residence**		
Urban	184	76.7
Rural	56	23.3
**Marital status**		
Single	21	8.8
Married	139	57.9
Divorced	34	14.2
Widowed	46	19.2
**Clinical characteristics**		
**Treatment**^**c**^		
Surgery	201	33.4
Radiotherapy	72	12
Chemotherapy	228	37.9
Hormonal therapy	101	16.8
**Stage of tumour**		
Stage I	23	9.6
Stage II	87	36.3
Stage III	98	40.8
Stage IV	32	13.3
**Admission status**		
New	8	3.3
Followup	232	96.7
**Commorbidty**		
Yes	39	16.3
No	201	83.8

*SD* Standard deviation

^a^Jehovah’s witnesses

^b^Private organisation

^c^The sum of frequency is greater than total because it is multiple response items

### Descriptive statistics of quality of life

#### EORTC QLQ-C30

The mean score of GHS/QoL of study participants was 65.6 (SD ± 18.64).

The mean score for EORTC QLQ-C30 functioning scales was reported as physical (69.9, SD ± 19.1), role (79.7, SD ± 26.9), emotional (73.3, SD ± 25.6), cognitive (77.4, SD ± 23.6) and social functioning (68.9, SD ± 28.4).

The highest mean score on the symptom scales/items were financial difficulties (50, SD ± 38.6), appetite loss (37.4, SD ± 36.4) and fatigue (34.3, SD ± 27.1), while the lowest symptom scores was diarrhoea (6.4 ± 18.4). Physical function, cognitive function, social function, fatigue, nausea vomiting, pain, insomnia, appetite loss, constipation, diarrhoea, and financial difficulties indicated lower QoL mean score than the EORTC QLQ-C30 reference values. The reference mean score values for each scales include: physical function (78.4), cognitive function (81.5), social function (77.0), fatigue (33.3), nausea vomiting (7.7), pain (28.7), insomnia (29.8), appetite loss (18.5), constipation (17.4), diarrhoea (5.9), and financial difficulties (18.3). Reference values provide data about the distribution of QoL scores for given cancer populations to compare a group of patients with similar characteristics, and to explain differences in clinical outcomes [23].

#### EORTC QLQ-BR45

The EORTC QLQ-BR45 results showed that, the mean score for functional scales ranged from future perspective (57.1, SD ± 37.3) to sexual enjoyment score (85.5, SD ± 26.3). The most affected functional scale was future perspective (57.1 ± 37.3). Upset by hair loss was the most affected symptom scale with mean score of (41.8, SD ± 34.6), majority of the participants (65.4%) were upset by hair loss. The endocrine sexual scale was the least affected symptom scale (mean = 7.2, SD ± 14.7), as only 26.2% of the participants had an affected endocrine sexual scale (Table 2).

**Table 2:** Mean and standard deviation of EORTC QLQ-C30 & BR45 components for breast cancer patients at TASH, Addis Ababa, Ethiopia, 2021

EORTC QLQ-C30	Items	Mean ± SD	EORTC QLQ-BR45	Items	Mean ± SD
**Functional scale**			**Functional scale**		
Physical function (PF)	1–5	69.9 **±** 19.1	Body image (BRBI)	9–12	78.64 **±** 25.9
Role function (RF)	6, 7	79.7 **±** 26.9	Sexual functioning (BRSEF)	14, 15	85.4 **±** 22.0
Emotional function (EF)	21–24	73.3 **±** 25.6	Sexual enjoyment (BRSEE)	16	85.5 **±** 26.3
Cognitive function (CF)	20, 25	77.4 **±** 23.6	Future perspective (BRFU)	13	57.1 **±** 37.3
Social function (SF)	26, 27	68.9 **±** 28.4	Breast satisfaction (BRBS) †	44, 45	71 **±** 29.0
Global Health Status (GHS)	29, 30	65.6 **±** 18.6			
**Symptom scale**			**Symptom scale**		
Fatigue (FA)	10, 12,18	34.3 **±** 27.1	Systemic therapy side effects (BRST)	1–4, 6, 7, 8	29 **±** 20.0
Nausea and vomiting (NV)	14, 15	23.8 **±** 32.5	Breast symptoms (BRBS)	20–23	19.95 **±** 25.3
Pain (PA)	9, 19	29.7 **±** 27.1	Arm symptoms (BRAS)	17, 18, 19	22.7 **±** 23.0
Dyspnea (DY)	8	16.1 **±** 23.2	Upset by hair loss (BRHL)	5	41.8 **±** 34.6
Insomnia (SL)	11	30.4 **±** 33.2	Endocrine therapy scale (BRET)	2 4–26,33–39	17.8 **±** 16.0
Appetite loss (AP)	13	37.4 **±** 36.4	Endocrine sexual scale (BRES)	40–43	7.2 **±** 14.7
Constipation (CO)	16	23.6 **±** 31.0	Skin mucosis scale (BRSM)	27–32	14.3 **±** 15.5
Diarrhoa (DI)	17	6.4 **±** 18.4			
Financial difficulties (FI)	28	50 **±** 38.6			

An increased age was significantly associated with higher body image (*P =* 0.003), sexual functioning (*P <* 0.001), sexual enjoyment (*P =* 0.001) and future perspective (*P =* 0.039). An increased age was associated with lower cognitive function (*P <* 0.001). Tumor stage had no significant association with functional scores of EORTC QLQ-C30 (Table 3 and Table S1).

**Table 3:** EORTC QLQ-C30 functional scores by socio-demographic and clinical characteristics among breast cancer patients, at TASH, Addis Ababa, Ethiopia, 2021

Variables	GHS/ QoL	Physical function	Role function	Emotional function	Cognitive function	Social function
	Mean ± SD	Mean ± SD	Mean ± SD	Mean ± SD	Mean ± SD	Mean ± SD
**Age**						
≤36	65.5 ± 18.0	72.2 ± 17.9	80.7 ± 25.7	69.6 ± 24.7	80.6 ± 21.8	66.1 ± 28.1
37–42	66.5 ± 19.8	75.6 ± 17.2	79.6 ± 25.4	74.0 ± 23.7	84.8 ± 22.4	71.0 ± 25.9
43–50	65.1 ± 17.3	67.1 ± 21.0	78.6 ± 30.4	70.8 ± 30.5	76.7 ± 23.0	65.5 ± 31.3
> 50	65.3 ± 20.0	64.7 ± 18.6	80.0 ± 26.7	78.6 ± 22.6	67.5 ± 24.4	73.3 ± 27. 8
*p* value	0.978	**0.007**	0.981	0.218	**< 0.001**	0.363
**Educational status**						
No education	67 ± 17.8	65.8 ± 20.1	76.2 ± 28.9	76.7 ± 25.2	72.7 ± 22.9	68.4 ± 30.6
Primary education	64.7 ± 17.1	65.4 ± 20.1	75.5 ± 28.5	65.8 ± 26.2	75.8 ± 24.1	65.7 ± 26.8
Secondary education	63.6 ± 19.6	72.8 ± 17.3	80.1 ± 27.2	74.1 ± 26.6	79.5 ± 24.8	68.7 ± 28.9
Above secondary education	66.5 ± 20.3	76.2 ± 16.5	87.7 ± 20.8	74.4 ± 24.0	82.7 ± 22.3	72.8 ± 26.5
*p* value	0.570	**0.002**	0.055	0.123	0.085	0.627
**Occupational status**						
House wife	65.4 ± 18.8	66.6 ± 19.5	77.9 ± 27.0	73.1 ± 25.9	75.4 ± 24.0	70.6 ± 26.3
Government employed	66.0 ± 22.4	75.0 ± 18.9	86.2 ± 25.1	80.7 ± 20.3	83.3 ± 20.2	70.0 ± 30.2
Farmer	72.7 ± 12.2	64.8 ± 20.6	70.0 ± 30.0	69.3 ± 28.5	71.3 ± 23.8	58.7 ± 37.9
Merchant	60.6 ± 19.3	77.8 ± 9.0	81.1 ± 21.7	69.4 ± 24.9	81.1 ± 21.7	64.4 ± 22.6
Daily labourer	62.8 ± 9.4	76.4 ± 13.2	92.3 ± 16.1	72.4 ± 18.1	84.6 ± 20.9	65.4 ± 28.4
Other	62.3 ± 20.6	80.4 ± 16.1	84.3 ± 32.0	68.1 ± 33.4	80.4 ± 29.0	75.5 ± 28.9
*p* value	0.355	**0.003**	0.098	0.473	0.250	0.384
**Residence**						
Urban	63.6 ± 19.8	71.2 ± 18.3	80.9 ± 26.6	72.8 ± 24.9	78.1 ± 23.9	70.4 ± 26.6
Rural	71.0 ± 13.1	65.6 ± 21.0	75.9 ± 27.9	74.6 ± 28.0	75.0 ± 22.7	64.3 ± 33.4
*p* value	**0.013**	0.056	0.225	0.660	0.395	0.160
**Marital status**						
Single	68.7 ± 21.1	72.7 ± 20.1	78.6 ± 23.1	76.2 ± 25.6	74.6 ± 27.2	69.1 ± 30.4
Married	66.3 ± 18.5	71.8 ± 19.0	80.6 ± 27.3	71.8 ± 27.0	80.5 ± 23.1	66.7 ± 28.7
Divorced	61.8 ± 18.5	67.8 ± 19.0	76.0 ± 30.2	70.1 ± 26.7	77.9 ± 21.6	67.2 ± 26.4
Widowed	64.9 ± 18.3	64.3 ± 18.5	80.4 ± 25.7	78.4 ± 19.8	68.8 ± 23.5	77.2 ± 27.3
*p* value	0.520	0.109	0.836	0.377	**0.033**	0.179
**Stage of tumour**						
Stage I	69.2 ± 21.1	73.9 ± 20.5	80.4 ± 27.8	69.2 ± 29.5	76.1 ± 27.0	74.6 ± 27.5
Stage II	65.8 ± 18.2	70.3 ± 17.8	81.8 ± 25.8	74.1 ± 26.8	79.1 ± 21.8	71.5 ± 27.8
Stage III	65.7 ± 18.8	70.1 ± 18.5	79.9 ± 26.4	74.3 ± 24.5	76.2 ± 23.9	67.9 ± 27.3
Stage IV	62.0 ± 17.8	64.8 ± 22.9	72.9 ± 31.0	70.3 ± 23.7	77.1 ± 26.0	61.5 ± 32.9
*p* value	0.559	0.340	0.463	0.739	0.853	0.265
**Comorbidity**						
No	66.5 ± 18.4	72.4 ± 17.7	81.0 ± 25.2	74.7 ± 24.6	79.0 ± 22.9	71.1 ± 27.3
Yes	61.1 ± 19.3	56.9 ± 21.0	73.1 ± 34.1	65.6 ± 30.0	68.8 ± 25.7	58.1 ± 31.7
*P* value	0.101	**< 0.001**	0.092	0.042	**0.013**	**0.009**

Those patients who had above secondary educational level had significantly a higher score for a physical function (*P =* 0.002). Those who lived in rural area had higher GHS/QoL scale than those who lived in urban area (*P =* 0.013). Farmers had experienced more nausea and vomiting (*P =* 0.044) as compared to others.

Married study participants had better cognitive function than participants with single, divorced and widowed marital status (*P =* 0.033). Absence of comorbidity was associated with better physical function (*P <* 0.001), cognitive function (*P =* 0.013), and social function (*P =* 0.009) (Table 3).

Age had no association with EORTC QLQ-C30 symptom scales. Whereas, patients who had comorbidity had more insomnia (*P =* 0.029), appetite loss (*P =* 0.014) and diarrhoea (*P =* 0.007). Presence of stage IV tumor was associated with more pain (*P =* 0.041) and appetite loss (*P =* 0.042). Educational status had a significant association with financial difficulties (*P =* 0.005) (Table 4).

**Table 4:** EORTC QLQ-C30 symptom scores by socio-demographic and clinical characteristics among breast cancer patients in TASH, Addis Ababa, Ethiopia, 2021

Variables	Fatigue	Nausea and Vomiting	Pain	Dyspnoea	Insomnia	Appetite loss	Constipation	Diarrhoea	Financial difficulties
	Mean ± SD	Mean ± SD	Mean ± SD	Mean ± SD	Mean ± SD	Mean ± SD	Mean ± SD	Mean ± SD	Mean ± SD
**Age**									
≤36	38.8 ± 25.3	25.0 ± 32.5	30.4 ± 24.6	12.9 ± 21.2	26.8 ± 32.4	35.5 ± 33.5	27.9 ± 30.3	5.4 ± 13.7	60.2 ± 37.6
37–42	29.1 ± 25.2	22.1 ± 30.2	29.3 ± 28.8	14.9 ± 25.1	32.7 ± 32.1	37.9 ± 38.7	18.4 ± 30.7	3.4 ± 14.9	44.3 ± 37.1
43–50	35.5 ± 28.5	28.8 ± 36.7	30.8 ± 29.1	15.5 ± 21.7	36.7 ± 35.1	35.5 ± 36.2	23.8 ± 30.7	10.5 ± 24.9	47.2 ± 39.4
> 50	33.3 ± 29.0	19.2 ± 30.4	28.3 ± 26.3	21.1 ± 24.5	25.5 ± 32.7	40.5 ± 37.8	23.8 ± 32.5	6.1 ± 17.8	47.7 ± 39.0
*p* value	0.252	0.406	0.959	0.247	0.222	0.854	0.416	0.192	0.104
**Educational status**									
No education	35.5 ± 28.1	23.1 ± 31.1	30.6 ± 27.6	22.6 ± 26.9	31.1 ± 35.2	40.0 ± 36.7	21.3 ± 27.7	6.66 ± 17.3	57.7 ± 38.4
Primary education	38.9 ± 28.6	28.4 ± 37.0	34.9 ± 29.6	15.0 ± 22.4	33.3 ± 34.6	40.5 ± 36.7	20.9 ± 30.5	9.80 ± 25.2	55.5 ± 38.1
Secondary education	33.7 ± 28.2	24.3 ± 33.1	32.2 ± 28.3	12.3 ± 18.5	32.2 ± 31.5	33.9 ± 34.2	29.3 ± 35.6	4.09 ± 16.7	49.7 ± 38.8
Secondary education and above	29.0 ± 22.8	20.2 ± 29.8	21.3 ± 20.5	12.3 ± 21.5	25.1 ± 31.0	34.5 ± 38.3	23.4 ± 30.8	5.31 ± 13.7	35.0 ± 35.3
*p* value	0.278	0.621	**0.047**	**0.026**	0.571	0.654	0.449	0.416	**0.005**
**Occupational Status**									
House’s wife	36.3 ± 26.9	20.8 ± 30.7	31.6 ± 27.7	15.5 ± 23.3	30.4 ± 34.1	34.3 ± 35.3	26.9 ± 32.7	7.40 ± 21.4	51.8 ± 38.7
Government employed	29.8 ± 24.6	19.0 ± 29.4	20.5 ± 19.8	9.5 ± 19.1	26.6 ± 32.1	30.5 ± 36.5	20.9 ± 30.3	3.80 ± 13.5	39.0 ± 35.6
Farmer	38.6 ± 30.7	38.6 ± 34.6	36.6 ± 30.0	28.0 ± 22.9	42.6 ± 34.0	53.3 ± 31.9	18.6 ± 23.7	9.33 ± 18.0	60.0 ± 36.0
Merchant	37.7 ± 33.8	34.4 ± 38.5	28.8 ± 29.8	13.3 ± 21.	26.6 ± 22.5	42.2 ± 38.7	24.4 ± 29.5	4.44 ± 11.7	48.8 ± 43.4
Daily laborer	23.1 ± 19.5	14.1 ± 24.4	21.8 ± 22.9	15.4 ± 22.0	30.7 ± 28.7	48.7 ± 35.0	15.4 ± 22.0	2.56 ± 9.24	48.7 ± 37.5
Others	26.8 ± 24.5	33.3 ± 41.6	30.4 ± 27.7	19.6 ± 29.1	23.5 ± 36.8	39.2 ± 46.0	15.6 ± 35.5	3.92 ± 11.1	45.1 ± 42.4
*p* value	0.297	**0.044**	0.184	0.072	0.447	0.126	0.491	0.740	0.404
**Residence**									
Urban	34.5 ± 26.9	21.6 ± 31.7	29.2 ± 27.0	14.9 ± 23.3	29.5 ± 32.9	35.7 ± 36.6	25.2 ± 32.5	6.0 ± 19.3	47.8 ± 38.7
Rural	33.5 ± 27.8	31.3 ± 34.5	31.5 ± 27.5	20.2 ± 22.6	33.3 ± 34.2	42.9 ± 35.8	18.5 ± 25.4	7.7 ± 15.6	57.1 ± 37.5
*P*-value	0.808	0.051	0.566	0.129	0.454	0.198	0.156	0.533	0.114
**Marital status**									
Single	36.0 ± 28.1	30.2 ± 33.2	28.6 ± 24.0	11.1 ± 21.9	25.4 ± 31.5	40.0 ± 34.3	30.2 ± 34.8	6.3 ± 13.4	54.0 ± 40.1
Married	31.3 ± 26.5	23.0 ± 31.7	27.3 ± 28.3	14.1 ± 21.6	29.3 ± 33.0	33.6 ± 35.5	22.1 ± 30.0	6.5 ± 18.8	47.0 ± 38.2
Divorced	40.0 ± 27.0	27.5 ± 38.0	39.2 ± 29.3	9.8 ± 19.3	30.4 ± 31.1	46.1 ± 37.6	16.7 ± 28.7	5.9 ± 19.2	52.9 ± 40.3
Widowed	38.4 ± 28.4	21.0 ± 30.7	30.4 ± 21.7	29.0 ± 27.0	36.2 ± 36.4	41.3 ± 38.6	30.4 ± 33.6	6.5 ± 18.4	55.1 ± 38.0
*P*-value	0.238	0.646	0.149	**< 0.001**	0.563	0.254	0.158	0.999	0.564
**Stage of tumor**									
Stage I	35.2 ± 22.1	21.7 ± 31.5	31.2 ± 27.7	7.24 ± 14.05	18.8 ± 28.1	27.5 ± 32.8	14.5 ± 24.3	11.6 ± 25.8	44.9 ± 42.2
Stage II	33.1 ± 26.3	21.6 ± 30.7	25.1 ± 23.1	18.3 ± 24.3	33.3 ± 35.2	34.8 ± 38.0	22.9 ± 29.3	6.5 ± 18.9	45.9 ± 37.4
Stage III	32.8 ± 27.9	22.3 ± 32.6	30.1 ± 27.5	15.6 ± 23.0	31.6 ± 32.9	37.1 ± 34.8	23.1 ± 31.2	5.10 ± 16.8	52.7 ± 38.5
Stage IV	41.3 ± 30.03	35.9 ± 36.4	40.10 ± 33.02	17.7 ± 25.4	27.1 ± 31.0	52.1 ± 36.8	33.3 ± 37.8	6.25 ± 15.6	56.3 ± 39.2
*P*-value	0.456	0.162	**0.041**	0.223	0.271	**0.042**	0.159	0.512	0.447
**Comorbidity**									
No	33.7 ± 27.1	22.7 ± 31.7	28.9 ± 26.4	15.9 ± 23.6	28.4 ± 32.1	34.8 ± 36.3	24.5 ± 31.8	4.97 ± 16.6	49.1 ± 39.2
Yes	37.0 ± 27.5	29.5 ± 36.2	33.7 ± 30.5	17.1 ± 21.5	41.0 ± 37.0	50.4 ± 34.9	18.8 ± 26.3	13.6 ± 25.0	54.7 ± 35.4
*P*- value	0.493	0.235	0.310	0.773	**0.029**	**0.014**	0.292	**0.007**	0.407

There were no significant association between comorbidity and breast cancer specific functional and symptom scales. Stage IV breast cancer patients had more arm symptoms (*P =* 0.003) compared to other stages. An increased age was associated with lower upset by hair loss (*P =* 0.001) (Table S1).

### EORTC-QLQ C30

The linear regression analysis showed the predictors association with the functional and symptom scales of EORTC QLQ-C30.

The predictors explained variations in physical functioning (R^2^ = 0.135), role functioning (R^2^ = 0.005), emotional functioning (R^2^ = 0.015), and social functioning (R^2^ = 0.027) (Table S2).

Patients with comorbidity, had lower physical functioning (B = − 0.278, *p =* 0.001), emotional functioning (B = − 0.138, *p =* 0.038), and social functioining (B = − 0.157, *p =* 0.018) than those who had no comorbidity.

Patients with secondary education and above had more role functioning (B = 0.198, *p =* 0.015) than those who had no education. Patients with stage IV breast cancer had lower physical functioning (B = − 0.174, *p =* 0.050) than those who with stage I breast cancer (Table S2).

Regarding EORTC QLQ-C30 symptom scales, stage IV breast cancer patients had more appetite loss (B = 0.245, *p =* 0.009) and constipation (B = 0.191, *p =* 0.044) than stage I breast cancer patients. In addition, patients with comorbidity had more appetite loss (B = 0.169, *p =* 0.011) than those who had no comorbidity (Table S3).

### EORTC-QLQ BR45

The predictors explained variations from (R^2^ = 0.026) to (R^2^ = 0.213) in breast satisfaction and sexual functioning scales. In multivariable linear regression model, stage IV breast cancer patients had lower sexual functioning (B = − 0.184, *p =* 0.030) and sexual enjoyment (B = − 0.184, *p =* 0.041) than stage I breast cancer patients.

Those participants aged above 50 had lower body image (B = − 0.238, *p =* 0.008), sexual functioning (B = − 0.249, *p =* 0.002), sexual enjoyment (B = − 0.216, *p =* 0.012), and future presepctive (B = − 0.212, *p* = 0.018) than participants aged ≤36. Married participants had better sexual functioning (B = 0.267, *p* = 0.012), and sexual enjoyment (B = 0.285, *p* = 0.011) than participants with single marital status (Table 5).

**Table 5:** Linear regression model with parameter estimates for the EORTC QLQ-BR45 functioning scales among breast cancer patients in TASH, Addis Ababa, Ethiopia, 2021

	Body image		Sexual functioning		Sexual enjoyment		Future perspective		Breast satisfaction	
	B (95% CI) R^2^ = 0.031	*p* value	B (95% CI) R^2^ = 0.213	*p* value	B (95% CI) R^2^ = 0.111	*p* value	B (95% CI) R^2^ = 0.047	*p* value	B (95% CI) R^2^ = 0.026	*p* value
**Stage of tumor**										
Stage I	REF		REF		REF		REF		REF	
Stage II	−.050 (− 14.8–9.4)	0.661	.136 (− 3.1–15.4)	0.190	.132 (− 4.5–19.0)	0.229	−.074 (−22.1–12.6)	0.512	−.061(− 0.3–0.5)	0.594
Stage III	−.096 (− 17.0–6.8)	0.402	.069 (− 6.0–12.2)	0.507	.128 (− 4.7–18.4)	0.243	−.061 (− 20.7–13.4)	0.595	−.200 (− 0.1–0.8)	0.084
Stage IV	−.071 (− 19.4–8.5)	0.445	−.184 (1.1–22.6)	**0.030**	−.184 (0.6–27.8)	**0.041**	−.071 (− 26.7–13.5)	0.444	−.028 (− 0.4–0.5)	0.766
**Comorbidity**										
No	REF		REF		REF		REF		REF	
Yes	−.016 (− 10.2–8.0)	0.809	.091(− 1.6–12.3)	0.128	.040 (− 6.0–11.6)	0.530	−.025 (− 15.7–10.5)	0.704	−.030 (− 0.2–0.4)	0.648
**Marital status**										
Single	REF		REF		REF		REF		REF	
Married	−.020 (−13.1–11.0)	0.861	.267 (− 21.1–2.7)	**0.012**	.285 (− 26.8–3.4)	**0.011**	.030 (− 14.9–19.3)	0.796	−.188 (− 0.7–0.1)	0.108
Divorced	−.044 (− 17.8 – 11.3)	0.661	.121 (− 3.5–18.7)	0.178	−.029 (− 16.3–12.0)	0.757	−.001 (− 20.8–21.0)	0.995	−.201(−1.0–0.1)	**0.045**
Widowed	.034 (− 12.0–16.5)	0.755	−.005(− 11.2–10.6)	0.958	−.094 (− 20.2–7.6)	0.372	.150 (− 6.2–34.5)	0.172	.178 (− 0.9–0.1)	0.109
**Educational status**										
No education	REF		REF		REF		REF		REF	
Primary education	.065 (−5.4–13.6)	0.395	.007 (− 6.9–7.6)	0.925	−.046 (− 12.2–6.3)	0.533	−.058 (− 18.9–8.3)	0.443	−.011(− 34.3–0.3)	0.888
Secondary education	− 078 (− 14.3–4.7)	0.327	.046 (− 4.9–9.6)	0.522	−.020 (− 10.5–8.0)	0.796	−.122 (− 24.3–2.9)	0.123	−.080 (− 0.5–0.2)	0.313
Secondary education and above	−.099 (− 15.6–3.6)	0.219	−.131 (− 14.1–0.6)	0.071	−.138 (− 17.8–0.8)	0.074	−.067 (− 19.6–7.8)	0.398	−.107 (− 0.5–0.1)	0.185
**Age**										
≤ 36	REF		REF		REF		REF		REF	
37–42	.146 (0.6–18.2)	0.067	.055 (− 4.4–10. 0)	0.444	.037 (− 6.9–11.4)	0.626	.117 (− 3.3 – 23.6)	0.139	−.095 (− 0.5–0.1)	0.233
43–50	.160 (0.1–19.1)	**0.048**	.116 (− 1.4–13.1)	0.113	.151(− 0.1–18.4)	**0.052**	.087 (− 6.1 - 21.0)	0.281	.039 (− 0.2–0.4)	0.626
> 50	−.238 (3.7–24.7)	**0.008**	−.249 (4.5–20.7)	**0.002**	−.216 (2.9–23.4)	**0.012**	−.212 (3.2–33.3)	**0.018**	.040 (− 0.3–0.4)	0.658

*REF* Reference category

The predictors explained variations in breast symptom (R^2^ = 0.075), and upset by hair loss (R^2^ = 0.133). Married and divorced participants were less upset by hair loss (B = − 0.443, *p* = 0.015), (B = − 0. 302, *p* = 0.047) respectively as compared to participants with single marital status. Participants aged above 50 years had higher score in breast symptom scale (B = 0.298, *p* = 0.001), and lower score in upset by hair loss scale (B = − 0.344, *p* = 0.002) (Table 6).

**Table 6:** Linear regression model with parameter estimates for the EORTC QLQ-BR45 symptom scales among breast cancer patients in TASH, Addis Ababa, Ethiopia, 2021

	Breast symptom		Upset by hair loss	
	B (95% CI) R^2^ = 0.075	*p* value	B (95% CI) R^2^ = 0.133	*p* value
**Stage of tumor**				
Stage I	REF		REF	
Stage II	−.176 (−20.8–2.3)	0.116	.287(−0.3–41.7)	0.053
Stage III	−.049 (− 13.9–8.8)	0.662	.269 (− 1.0–39.5)	0.062
Stage IV	.031 (− 11.1–15.7)	0.773	.170 (− 7.6–37.6)	0.191
**Comorbidity**				
No	REF		REF	
Yes	−.062 (−4.4–13.0)	0.333	.126 (− 4.1–10.5)	0.150
**Marital status**				
Single	REF		REF	
Married	−.089 (−16.0 – 6.9)	0.433	−.443 (− 57.1–6.2)	**0.015**
Divorced	.078 (− 8.2 – 19.5)	0.425	−.302 (− 59.3–0.383)	**0.047**
Widowed	−.014 (− 14.5–12.7)	0.897	−.292 (− 56.0–2.6)	0.074
**Educational status**				
No education	REF		REF	
Primary education	.015 (− 8.1–10.0)	0.837	−.059 (− 21.3–11.3)	0.545
Secondary education	−.134 (− 17.0–1.1)	0.086	.110 (−6.6–24.0)	0.267
Secondary education and above	−.146 (− 17.8–0.50)	0.064	.139 (− 5.1–28)	0.171
**Age**				
≤ 36	REF		REF	
37–42	.225 (− 22.3–4.2)	**0.004**	−.136 (− 26.5 – 5.0)	0.178
43–50	.234 (22.7–2.0)	**0.003**	−.283 (− 38.6 – 6.7)	**0.006**
> 50	.298 (27.5–7.3)	**0.001**	−.344 (− 47.3–11.0)	**0.002**

*REF* Reference category

## Discussion

The aim of this study was to assess quality of life and factors affecting it among breast cancer patients in Addis Ababa using the newly updated breast cancer-specific (EORTC QLQ-BR45) and general (EORTC QLQ-C30) instruments. The main findings in this study were: (1) EORTC QLQ-C30 and BR45 functioning and symptoms scales showed financial difficulties, fatigue, loss of appetite, social functioning, future perspective and upset by hair loss were the most affected EORTC QLQ scales among breast cancer patients (2) Factors associated to QoL of breast cancer patients were age, stage of tumor, educational status, comorbidity, and place of residence.

### EORTC QLQ-C30 functioning and symptom scales

The mean GHS/QoL score of our study was 65.6. This value is similar to studies from Malaysia and Morocco conducted on quality of life in breast cancer patients [24, 25]. In these studies the value was 69.12 [24] and 68.5 [25]. These values can be explained by similarity of participant’s stage of disease among these studies. However, the GHS/QoL mean score in this study was higher than the EORTC QLQ-C30 reference value (GHS/QoL mean = 61.8, SD = 24.6) [23]. A study conducted in China [9] among breast cancer patients in which a larger proportion of patients who had received chemotherapy. This disparity can be explained by the fact that patients receiving chemotherapy may have multiple side effects that have a negative impact on GHS/QoL. Social function was the most affected scale, similar to previous studies conducted in Ethiopia [13] and China [9]. The reduction of social functioning in this study might reflect insufficient social support for the patients. In addition, most of the participants in this study were housewives who spend most of their time in the house that could affect their social interaction. Regarding symptom scales, severe impairment was observed in terms of financial difficulties (50, SD ± 38.6), appetite loss (37.4, SD ± 36.4), and fatigue (34.3, SD ± 27.1). This finding was consistent with other previous studies conducted in Ethiopia [13], China [9], Morocco [25], Egypt [26], Nepal [27], Malaysia [24], and Brazil [28]. As we see this can be explained by treatment side effects, which causes various symptoms like fatigue and appetite loss. The majority of the participants were in advanced stages, where it is common for such symptoms to occur. In addition, poor economic status, inability to work, and medical expenses can result in financial difficulties [11, 28]. Similarly, fatigue was the highest symptom scale among Brazilian breast cancer survivors [29] and Saudi Arabian breast cancer patients [30]. Moreover, financial difficulties were the least disturbing symptom among Saudi Arabian [30], Sweden [31], and Brazilian [28] breast cancer patients. The difference can be explained by difference in the economic status of the countries and Ethiopian population is categorized as low-income country. The majority of the Ethiopian participants were jobless. Thus, in addition to the usual household expenses, the medical expenses could have increased financial difficulties.

In our study, the EORTC QLQ-BR45 functional scale future perspective (mean = 57.1, SD ± 37.3) was the most affected scale. This result is in line with studies from Morocco [25] and China [9].

However, this finding contrasts to a study done in Kuwait, where about two-thirds of the participants were optimistic about their future health [32]. This difference in future perspective could be attributed to a lack of awareness about the disease and its treatment, associated stigma and sense of hopelessness, and the lengthy referral system to the country’s only oncology centre [33]. Regarding symptom scales, being upset by hair loss (41.8, SD ± 34.6) was the most affected scale, similar with other studies [9, 29]. This may be due to the young average age of the participants in this study; additionally, most of the participants had received chemotherapy, which often resulted in hair loss. Hair loss is one of the most common side effects of chemotherapy.

In contrast, the study from China showed that breast symptoms was the most affected scale [9]. This disparity could be due to the varying types of cancer treatment. In this study, the different treatment types for cancer included chemotherapy, which alone contributed to hair loss, whereas in the latter study, surgery was the only type of treatment among participants. Consistent with a previous study conducted in Morocco [25], participants in the younger age group had significantly lower body image (*P =* 0.040), sexual functioning (*P =* 0.001), sexual pleasure (*P =* 0.007), and future prospects (*P =* 0.039). However, one explanation could be that physical appearance, sexual functioning, and sexual pleasure are more important at younger ages and that women whose body image has changed because of hair loss and surgical procedures may feel emotionally depressed, which may prevent them from participating in sexual and social activities.

### Factors associated to QoL of breast cancer patients

As we know from a study in Turkey [34], tumor stage was significantly associated with pain (*p =* 0.041) and arm symptoms (*p =* 0.042). Stage IV breast cancer patients had lower physical functioning, sexual functioning, sexual enjoyment and more arm symptom as compared to stage I breast cancer patients. In line with this study, another study reported that patients with advanced stage of cancer had unfavorable QoL scales [35] and patients with early stage of cancer showed higher functional score and lower symptom score [36]. Age had no significant association with EORTC QLQ-C30 symptom scales. On contrary to our study, age had a significant association with Functional Assessment of Cancer Therapy-Breast questionnaire (FACT B-4) breast cancer QoL assessment tool [37]. This discrepancy between studies might be due to the variation in socioeconomic status, and different QoL assessment tools. In the present study, patients who had comorbidity had also more insomnia (*p =* 0.029), appetite loss (*p =* 0.014), and diarrhoea (*p =* 0.007). A study conducted in Cairo had showed patients who had no comorbidities had significantly higher social wellbeing (*p =* 0.014) and FWB (*p =* 0.004) scores than those who had comorbidities [37]. Similarly in our study, patients who had no comorbidity had better physical function (*p <* 0.001), cognitive function (*p =* 0.013), and social function (*p =* 0.009). A previous study conducted in America on the impact of comorbidity on QoL of cancer patients concluded that comorbidity exerts negative impacts on QoL of breast cancer patients [37–39]. This indicates that integrating information about comorbidity status to breast cancer care would improve the overall QoL outcomes of the patients by providing additional support and care for comorbidity. In this study from Ethiopia, educational status had no significant association with the GHS/QoL which is incongruent with a study conducted in Turkey [34]. However, advancement in the educational level of the participants showed a higher in physical function (*p =* 0.002) and less financial difficulties *(p =* 0.005). This might be explained by the fact that having better educational status could improve the health seeking behaviour of the patients (i.e. physical activity and nutritional modification) and it provides job opportunities that will be a source of income respectively.

All EORTC QLQ-C30 symptom scales except dyspnea indicated lower QoL of breast cancer patients as compared to EORTC QLQ-C30 reference values. This lower QoL scores we found among Ethiopian breast cancer patients could influence treatment adherence and patient’s survival. Therefore, it requires attention and immediate action to improve their QoL.

### Clinical implications

This study shows the need to improve QoL especially focusing on patients fatigue and financial difficulties. Clinicians need to focus on designing psychosocial interventions to reduce symptoms such as fatigue and support visions of future perspective to improve physical functioning, and QoL throughout their illness and treatment period. Planned education programs addressing patients’ needs, patient support and encouragement, financial aid, and incorporating group or individual psychosocial intervention into routine patient care are important interventions to consider for breast cancer patients.

In addition, our finding provided baseline information for future research on the QoL of Ethiopian breast cancer patients using a newly updated tool.

### Strengths and limitations of the study

This study is to our knowledge the first study conducted using a newly updated breast cancer specific QoL assessment tool (EORTC QLQ-BR45) in Ethiopia. The other strength of this study was a high response rate.

The limitation of the study included: a crosssectional design was utilized, which does not allow the detection change in QoL throughout the different phases of the disease. The average time since diagnosis which might have affected QoL was not included as an independent variable. The Amharic versions of EORTC QLQ-C30 and EORTC QLQ-BR45 were not tested for their responsiveness to change analysis during validity and reliability tests.

## Conclusion

Since our study showed considerable financial difficulties, social functioning from EORTC QLQ-C30 as well as impaired vision of future perspective among breast cancer patients in Ethiopia, efforts should be made to increase availability of social services at the tertiary referral center. Age, stage of tumor, educational status, comorbidity, and place of residence were important sociodemographic and clinical related factors associated with differences in QoL. Therefore, these specific groups of patients need additional attention about their ability to cope with the burden of the disease.

## Supplementary Information

**Additional file 1.**

## Data Availability

The dataset supporting the conclusions of this article is included within the article and its additional file(s).
