# Supplementary material for: Quality of life and its influencing factors among breast cancer patients at Tikur Anbessa specialised hospital, Addis Ababa, Ethiopia
Source: BMC Cancer. 2022 Aug 17;22:897. doi: 10.1186/s12885-022-09921-6 (PMC9382842; doi:10.1186/s12885-022-09921-6)
Supplement: Supplementary file 1 — Additional file 1. [file 12885_2022_9921_MOESM1_ESM.docx]

**Table S1.** EORTC QLQ-BR45 functional and symptom scores by socio-demographic and clinical characteristics among breast cancer patients, at TASH, Ethiopia, 2021.

| **Variables** | **Body image** | **Sexual functioning** | | **Sexual enjoyment** | | **Future perspective** | | **Systematic therapy side effects** | **Breast symptoms** | **Arm symptoms** | **Upset hair-loss** | **Endocrine therapy** | **Endocrine sexual** | **Skin mucosis** | **Breast satisfaction** |
| --- | --- | --- | --- | --- | --- | --- | --- | --- | --- | --- | --- | --- | --- | --- | --- |
| **Age**  ≤36  37-42  43-50  >50  *p* value | Mean ± SD | Mean ± SD | | Mean±SD | | Mean ± SD | | Mean ± SD | Mean ± SD | Mean ± SD | Mean ± SD | Mean ± SD | Mean ± SD | Mean ± SD | Mean ± SD |
|  | 70.0 ± 27.1  77.7 ± 24.5  80.1 ± 26.5  87.1 ± 22.7  **0.003** | 78.7 ± 25.1  81.3 ± 23.8  86.1 ± 21.3  95.5 ± 11.8  **0.000** | | 78.4 ± 29.6  79.8 ± 30.5  88.3 ± 24.4  95.5 ± 14.3  **0.001** | | 45.2 ± 34.7  56.3 ± 35.4  55.0 ± 39.2  72.2 ± 35.3  **0.039** | | 32.6 ± 20.3  25.6 ± 18.7  27.4 ± 18.9  30.2 ± 21.9  0.229 | 29.0 ± 29.2  16.5 ± 23.4  17.4 ± 21.3  16.5 ± 24.8  **0.012** | 26.8 ± 25.2  20.7 ± 21.7  20.4 ± 19.5  23.0 ± 25.2  0.379 | 57.3 ± 38.2  46.1 ± 31.8  31.3 ± 30.0  27.4 ± 28.8  **0.001** | 20.4 ± 13.8  16.6 ± 15.7  17.4 ± 15.3  16.4 ± 18.6  0.477 | 7.12 ± 12.5  9.33 ± 16.9  5.27 ± 11.6  6.94 ± 17.1  0.520 | 14.6 ± 15.1  15.6 ± 17.4  14.3 ± 16.4  12.8 ± 13.0  0.815 | 71.2±28.6  78.4 ±22.7  67.5 ± 29.9  66.1 ± 32.7  0.094 |
| **Educational status** | | | | | | | | | | | | | | | |
| No edu.  Primary educ.  Secondary educ.  Above secondary  *p* value | 82.7 ± 24.6  83.8 ± 20.1  73.8 ± 28.9  73.4 ± 27.7  **0.040** | | 90.4 ± 18.4  86.9 ± 23.2  87.4 ± 19.7  75.4 ± 24.6  **0.001** | 92.0 ± 21.1  85.6 ± 26.0  86.5 ± 26.6  76.0 ± 30.0  **0.007** | | 66.6 ± 36.4  55.5 ± 37.5  48.5 ± 38.8  54.4 ± 34.8  **0.039** | | 30.8 ± 20.7  30.6 ± 21.8  27.7 ± 17.7  26.5 ± 19.7  0.553 | 21.6 ± 28.8  24.6 ± 25.4  18.1 ± 23.7  15.4 ± 21.3  0.242 | 22.1 ± 23.1  25.1 ± 24.4  22.8 ± 23.8  21.6 ± 21.4  0.873 | 35.6 ± 34.4  33.3 ± 30.1  48.5 ± 37.3  51.2 ± 33.3  0.091 | 15.2 ± 15.0  20.0 ± 18.0  18.8 ± 15.2  17.9 ± 15.4  0.359 | 7.3 ± 15.6  9.2 ± 15.9  6.3 ± 14.4  5.9 ± 12.5  0.683 | 12.3 ± 14.7  15.1 ± 15.3  16.2 ± 18.3  14.43 ±13.5  0.525 | 67.1 ± 30.9  67.3 ± 27.8  73.1 ± 30.3  76.3 ± 25.4  0.225 |
| **Occupational status** | | | | | | | | | | | | | | | |
| House’s wife  Gov’t empl.  Farmer  Merchant  Laborer  Others  *p* value | 81.7 ± 23.5  75.5 ± 28.7  76.7 ± 31.5  68.3 ± 28.2  71.2 ± 26.7  77.9 ± 26.2  0.295 | | 88.4 ± 19.7  74.3 ± 26.3  89.3 ± 17.3  75.5 ± 23.5  93.6 ± 10.8  81.4 ± 30.5  0.003 | 87.2 ± 25.4  72.4 ± 32.8  93.3 ± 16.6  82.2 ± 24.7  97.4 ± 9.3  82.4 ± 31.4  **0.011** | | 60.0 ± 36.6  57.1 ± 36.6  52.0 ± 39.7  53.3 ± 41.4  53.8 ± 37.4  47.1 ± 39.2  0.739 | | 29.5 ± 20.4  26.1 ± 17.6  32.2 ± 20.4  26.3 ± 17.8  25.6 ±13.4  31.6 ± 27.5  0.801 | 19.7 ± 22.7  17.5 ± 24.8  24.4 ± 31.7  17.7 ± 29.9  13.6 ± 20.8  27.5 ± 34.5  0.622 | 22.6 ± 22.3  23.5 ± 24.1  27.5 ± 29.6  24.4 ± 21.9  10.3 ± 16.0  23.5 ± 21.5  0.413 | 35.7 ± 33.5  56.8 ± 40.4  39.2 ± 33.8  55.5 ± 33.3  43.3 ± 16.1  50.0 ± 42.3  0.183 | 18.0 ± 16.4  15.4 ± 13.5  19.2 ±18.5  19.3 ± 17.8  15.6 ±14.5  18.2 ± 13.9  0.928 | 6.9 ± 15.5  5.5 ± 13.1  14.3 ± 18.2  5.5 ± 8.1  5.1 ± 11.04  4.4 ± 9.4  0.188 | 13.3 ± 15.1  12.5 ± 12.5  16.0 ± 17.8  20.7 ± 19.4  18.4 ± 15.9  15.4 ± 16.0  0.426 | 70.1 ± 27.5  71.9 ± 29.1  72.0 ± 32.5  72.2 ± 33.1  84.6 ± 25.8  59.8 ± 32.8  0.350 |
| **Residence** | | | | | | | | | | | | | | | |
| Urban  Rural  *p* value | 77.8 ± 25.5  81.2 ± 27.3  0.392 | | 85.1 ± 21.7  86.6 ± 23.0  0.645 | 84.2 ± 27.2  89.8 ± 22.8  0.61 | | 55.4 ± 36.6  62.5 ± 39.2  0.215 | | 28.4 ± 20.1  31.0 ± 20.2  0.393 | 19.3 ± 24.6  22.0 ± 27.8  0.486 | 22.4 ± 22.8  24.0 ± 23.9  0.649 | 43.2 ± 34.6  37.9 ± 34.8  0.440 | 17.8 ±15.8  17.5 ±16.3  0.894 | 6.0 ± 14.1  10.8 ±16.0  **0.031** | 14.4 ± 15.2  13.9 ± 16.5  0.851 | 71.4 ± 28.2  68.7 ± 31.4  0.554 |
| **Marital status** | | | | | | | | | | | | | | | |
| Single  Married  Divorced  Widowed  *p* value | 76.1 ± 25.7  77.1 ± 26.5  76.7 ± 29.2  86.0 ± 20.6  0.198 | | 89.6 ± 19.3  78.1 ± 24.2  98.0 ± 6.8  96.0 ± 12.7  **0.000** | 93.6 ± 17.1  79.1 ± 29.1  93.1 ± 21.4  95.6 ± 18.1  **0.000** | | 49.2 ± 38.9  53.4 ± 36.0  52.9 ± 41.1  74.6 ± 33.1  **0.004** | 24.7 ± 17.8  29.4 ± 21.5  29.4 ± 18.8  29.5 ± 17.4  0.787 | | 22.75 ± 21.  17.9 ± 23.7  29.4 ± 30.4  17.6 ± 26.4  0.101 | 22.2 ± 18.6  21.2 ± 23.4  27.7 ± 21.9  24.2 ± 24.8  0.489 | 76.2 ± 41.8  41.2 ± 33.5  38.5 ± 35.6  36.2 ± 31.6  0.051 | 16.5 ±11.3  17.2 ±17.1  19.0 ±15.5  19.1 ±14.6  0.846 | 4.7 ± 10.7  7.7 ± 13.7  4.4 ± 11.0  8.3 ± 20.4  0.515 | 11.6 ± 10.4  14.5 ± 15.4  16.2 ± 20.2  13.4 ± 13.7  0.727 | 59.5 ± 28.2  71.9 ± 28.8  75.5 ± 24.7  68.8 ± 32.3  0.214 |
| **Stage** | | | | | | | | | | | | | | | |
| Stage I  Stage II  Stage III  Stage IV  *p* value | 80.1 ± 30.6  79.8 ± 24.7  78.6 ± 26.2  74.5 ± 25.4  0.780 | | 77.5 ± 27.8  84.8 ± 22.2  85.8 ± 21.5  91.1 ± 16.4  0.156 | 75.4 ± 33.6  84.6 ± 25.3  87.1 ± 26.1  90.6 ± 22.7  0.167 | | 59.4 ± 31.7  56.7 ± 38.1  58.5 ± 37.1  52.1 ± 40.5  0.847 | | 28.5 ± 22.5  28.7 ± 20.7  27.7 ± 20.1  33.7 ± 15.8  0.536 | 22.7 ± 24.5  15.3 ± 21.2  21.3 ± 27.2  26.4 ± 29.3  0.137 | 25.1 ± 24.6  18.7 ± 19.8  21.4 ± 21.3  36.1 ± 30.3  **0.003** | 28.2 ± 29.9  43.3 ± 37.4  42.8 ± 32.6  44.0 ± 35.6  0.529 | 16.6 ± 16.6  16.8 ± 15.0  18.3 ± 16.9  19.3 ± 15.2  0.855 | 8.7 ± 15.9  6.2 ± 13.1  6.1 ± 13.3  11.7 ± 20.5  0.244 | 15.7 ± 18.5  12.8 ± 12.8  13.7 ± 14.5  18.9 ± 21.2  0.278 | 78.9 ± 24.7  72.4 ± 29.2  65.5 ± 30.1  76.6 ± 26.0  0.083 |
| **Comorbidity** | | | | | | | | | | | | | | | |
| No  Yes  *p* value | 78.4 ± 25.3  79.9 ± 29.3  0.739 | 84.4 ± 22.3  90.6 ± 19.7  0.108 | | 84.7 ± 26.8  89.7 ± 23.1  0.278 | | 57.0 ± 36.2  57.2 ± 43.2  0.974 | | 28.0 ± 19.3  33.8 ± 23.2  0.103 | 19.3 ± 24.8  23.4 ± 27.7  0.360 | 22.49 ± 23.0  24.2 ± 23.2  0.671 | 41.5 ± 34.3  42.6 ± 36.6  0.889 | 17.5 ± 14.9  19.0 ± 20.4  0.576 | 6.9 ± 14.5  8.3 ± 15.6  0.585 | 13.8 ± 14.8  16.9 ± 18.5  0.249 | 71.7 ± 28.3  65.8 ± 32.4  0.245 |
| **Admission status** | | | | | | | | | | | | | | | |
| New  Followup  *p* value | 77.1 ± 29.7  78.7 ± 25.8  0.863 | 81.2 ± 24.3  85.5 ± 21.9  0.587 | | 66.6 ±39.8  86.2 ±25.6  **0.039** | 20.8 ± 24.8  58.3 ± 37.1  **0.005** | | | 27.9 ± 25.2  29.1 ± 19.9  0.880 | 26.4 ± 16.7  19.7 ± 25.5  0.466 | 34.7 ± 24.8  22.4 ± 22.9  0.137 | 22.2 ± 19.2  42.2 ± 34.7  0.324 | 19.1 ± 17.2  17.7 ± 15.9  0.799 | 12.5 ± 19.4  6.9 ± 14.5  0.296 | 14.5 ± 18.7  14.3 ± 15.41  0.962 | 68.7 ± 33.8  70.8 ± 28.9  0.842 |

^SD standard deviation^

**Table S2.** Linear regression model with parameter estimates for EORTC QLQ-C30 functioning scales among breast cancer patients, at TASH, Addis Ababa, Ethiopia, 2021.

|  | **Physical functioning** | | **Role functioning** | | **Emotional functioning** | | **Social functioning** | |
| --- | --- | --- | --- | --- | --- | --- | --- | --- |
|  | B (95 % CI)  R^2^ = 0.135 | *p* value | B (95 % CI)  R^2^=0.005 | *p* value | B (95 % CI)  R^2^=0.015 | *p* value | B (95 % CI)  R^2^=0.027 | *p* value |
| **Stage of tumor** |  |  |  |  |  |  |  |  |
| Stage I | REF |  | REF |  | REF |  | REF |  |
| Stage II | -.082 (-11.7 - 5.2 ) | 0.448 | .057 (-9.6 – 15.9 ) | 0.625 | .073 (-8.2 – 15.9 ) | 0.526 | -.074 (-17.7 – 9.0 ) | 0.516 |
| Stage III | -.041 (-9.9 – 6.7 ) | 0.707 | .039 (-10.4 - 14.6 ) | 0.740 | .092 (-7.1 – 16.6 ) | 0.428 | -.134 (-20.7 – 5.3 ) | 0.245 |
| Stage IV | -.174 (19.5 – 0.1 ) | **0.050** | -.087 (-21.6 – 7.8 ) | 0.358 | -.004 (-14.3 – 13.7 ) | 0.965 | -.173 (-29.8 - 0.9 ) | 0.065 |
| **Comorbidity** |  |  |  |  |  |  |  |  |
| No | REF |  | REF |  | REF |  | REF |  |
| Yes | -.278 (-20.7 - 8.0 ) | **0.001** | -.091 (-16.2 -3.0 ) | 0.174 | -.138 (-18.6- 0.5 ) | **0.038** | -.157 (-22.0 – 2.1 ) | **0.018** |
| **Marital status** |  |  |  |  |  |  |  |  |
| Single | REF |  | REF |  | REF |  | REF |  |
| Married | -.012 (-8.8 – 7.9) | 0.912 | .073 (-8.7 – 16.6 ) | 0.535 | -.073 (-15.8 – 8.2 ) | 0.534 | -.036 (-15 – 11.1 ) | 0.757 |
| Divorced | -.084(-14.7 – 5.5 ) | 0.371 | .018 (-14.0 - 16.7 ) | 0.858 | -.078 (-20.2 – 8.8 ) | 0.438 | -.012 (-16.9 – 15.0 ) | 0.905 |
| Widowed | -.124 (-16.0 – 4.0) | 0.236 | .063 (-10.8 – 19.3) | 0.576 | -.047 (-17.3 – 11.2 ) | 0.670 | .102 (-8.3 – 23.0 ) | 0.355 |
| **Educational status** |  |  |  |  |  |  |  |  |
| No education | REF |  | REF |  | REF |  | REF |  |
| Primary education | -.058 (-9.3 – 3.9 ) | 0.422 | -.006 (-10.4 – 9.6 ) | 0.939 | -.154 (-19.1 – 0.1 ) | **0.047** | .004 (-10.1 – 10.7 ) | 0.956 |
| Secondary education | .101 (-2.1 – 11.2 ) | 0.178 | .099 (-3.8 – 16.3 ) | 0.220 | -.000 (-9.5 – 9.5 ) | 0.995 | .067 (-6.0 – 14.8 ) | 0.402 |
| Secondary education and above | .123 (-1.2 – 12.2 ) | 0.105 | .198 (2.4 – 22.6 ) | **0.015** | -.011 (-10.3 – 8.9 ) | 0.889 | .095 (-4.2 – 16.9 ) | 0.237 |
| **Age** |  |  |  |  |  |  |  |  |
| ≤36 | REF |  | REF |  | REF |  | REF |  |
| 37-42 | .071 (-3.4 – 9.7 ) | 0.343 | -.038 (-12.3 – 7.5 ) | 0.635 | .070 ( -5.2 – 13.6 ) | 0.383 | .063 (-6.2 – 14.5) | 0.429 |
| 43-50 | -.062 (-9.3 – 3.9 ) | 0.416 | -.015 (-11.0 – 9.1 ) | 0.853 | .036 ( -7.4 - 11.6 ) | 0.660 | .001 (-10.4 – 10.5 ) | 0.991 |
| >50 | -.047 (-9.4 – 5.3 ) | 0.583 | .038 (-8.8 – 13.4 ) | 0.680 | .161 (-1.0 – 20.0 ) | 0.075 | .100 (-5.0 – 18.1 ) | 0.266 |

^REF Reference category^

**Table S3.** Linear regression model with parameter estimates for EORTC QLQ-C30 symptom scales among breast cancer patients, at TASH, Addis Ababa, Ethiopia, 2021.

^REF Reference category^

|  | **Diarrhoa** | | **Insomnia** | | **Appetite loss** | | **Constipation** | | **Financial difficulties** | |
| --- | --- | --- | --- | --- | --- | --- | --- | --- | --- | --- |
|  | B (95 % CI)  R^2^ = 0.013 | *p* value | B (95 % CI)  R^2^=0.027 | *p* value | B (95 % CI)  R^2^= 0.031 | *p* value | B (95 % CI)  R^2^= 0.015 | *p* value | B (95 % CI)  R^2^= 0.063 | *p*  value |
| **Stage of tumor** |  |  |  |  |  |  |  |  |  |  |
| Stage I | REF |  | REF |  | REF |  | REF |  | REF |  |
| Stage II | -.127 (-13.6 - 3.9 ) | 0.129 | .222 (-0.2 - 30.8 ) | 0.625 | .110 (-8.7 - 25.4 ) | 0.336 | .099 (-8.2 - 21.0 ) | 0.390 | -.071 (-23.4 - 12.0 ) | 0.530 |
| Stage III | -.185(-15.5 - 1.6 ) | 0.244 | .172 (-3.6 - 26.9 ) | 0.740 | .102 (-9.1 - 24.3 ) | 0.373 | .128 (-6.3 - 22.4 ) | 0.269 | .044 (-14.0 - 20.8 ) | 0.698 |
| Stage IV | .067 (-13.7 - 6.4 ) | 0.125 | .095 (-8.7 - 27.2 ) | 0.358 | .245 (6.4 - 45.9 ) | **0.009** | .191 (0.5 - 34.3 ) | **0.044** | .057 (-14.0 - 27.0 ) | 0.534 |
| **Comorbidity** |  |  |  |  |  |  |  |  |  |  |
| No | REF |  | REF |  | REF |  | REF |  |  |  |
| Yes | .156 (1.3 – 14.3 ) | 0.765 | .160 (2.7 - 26.0) | 0.174 | .169 (3.8 - 29.4 ) | **0.011** | -.063 (-16.3 – 5.7 ) | 0.346 | .046 (-8.5 -18.1 ) | 0.480 |
| **Marital status** |  |  |  |  |  |  |  |  |  |  |
| Single | REF |  | REF |  | REF |  | REF |  | REF |  |
| Married | -.023 (-9.5 – 7.8 ) | 0.651 | .060 (-11.4 – 19.5) | 0.535 | -.110 (-25.0 – 8.8 ) | 0.346 | -.114 (-21.7 – 7.4 ) | 0.333 | -.080 (-23.8 – 11.3 ) | 0.484 |
| Divorced | -.021(-11.6 – 9.3 ) | 0.731 | .056 (-13.3 - 24.0) | 0.858 | .047 (-15.6 – 25.3 ) | 0.639 | -.134 (-29.4 – 5.7 ) | 0.184 | -.034 ( -25.0 - 17.5 ) | 0.727 |
| Widowed | .013 (-9.6 – 10.9 ) | **0.015** | .194 (-2.0 - 34.7 ) | 0.576 | .004 (-19.7 – 20.5 ) | 0.968 | .051 (-13.3 – 21.2 ) | 0.650 | .047 (-16.3 – 25.4 ) | 0.665 |
| **Educational status** |  |  |  |  |  |  |  |  |  |  |
| No education | REF |  | REF |  | REF |  | REF |  | REF |  |
| Primary education | -.085 (-3.0 – 10.7 ) | 0.406 | .046 (-8.5 – 16.0 ) | 0.939 | .029 (-10 – 16.0 ) | 0.703 | .037 (-8.7 – 14.3 ) | 0.632 | -.032 (-17.0 -10.8 ) | 0.667 |
| Secondary education | -.055 (-9.2– 4.5 ) | 0.078 | .023 (-10.4 – 14.0 ) | 0.220 | -.069 (-19.3 – 7.5 ) | 0.385 | .109 (-3.6 – 19.4 ) | 0.176 | -.144 ( -27.0 – 0.9 ) | 0.067 |
| Secondary education and above | -.011 (-7.4 – 6.4 ) | 0.139 | -.036 (-15.1 – 9.5 ) | **0.015** | -.015 (-14.7 – 12.3 ) | 0.856 | .068 (-6.6 – 16.6 ) | 0.399 | -.277 ( -39.1 – 11.0 ) | **0.001** |
| **Age** |  |  |  |  |  |  |  |  |  |  |
| ≤36 | REF |  | REF |  | REF |  | REF |  | REF |  |
| 37-42 | -.031(-8.1 – 5.4 ) | 0.613 | .070 (-6.7 – 17.5 ) | 0.635 | .034 ( -10.4 – 16.1 ) | 0.670 | -.119 (-20.0 – 2.8) | 0.137 | -.174 (-29.4 -1.8 ) | **0.027** |
| 43-50 | .105 (-2.4– 11.3 ) | 0.833 | .076 (-6.4 – 18.1 ) | 0.853 | -.008 ( -13.4 – 13.0 ) | 0.925 | -.027 (-13.4 – 9.5 ) | 0.740 | -.198 (-31.5 -3.7 ) | **0.013** |
| >50 | -.019 (-8.4 – 6.8 ) | 0.864 | -.122 (-23.0 – 4.2 ) | 0.680 | --.003 (-15.0 – 14.6 ) | 0.978 | -.078 (-18.3 – 7.2 ) | 0.391 | -.280 (-40.3 – 9.4 ) | **0.002** |
